# Biomarker screening using integrated bioinformatics for the development of “normal—impaired glucose intolerance—type 2 diabetes mellitus”

**DOI:** 10.1038/s41598-024-55199-y

**Published:** 2024-02-24

**Authors:** Dongqiang Luo, Xiaolu Gao, Xianqiong Zhu, Jiongbo Xu, Pengfei Gao, Jiayi Zou, Qiaoming Fan, Ying Xu, Tian Liu

**Affiliations:** 1 Guangzhou University of Chinese Medicine, Guangzhou, 510000 China; 2 Yunkang School of Medicine and Health, Nanfang College Guangzhou, Guangzhou, 510000 China; 3 Foshan Hospital of Traditional Chinese Medicine, Foshan, 528000 China

**Keywords:** Type 2 diabetes, Developmental process, Impaired glucose tolerance, Bioinformatics, Oxidative stress, Computational biology and bioinformatics, Molecular biology, Endocrinology

## Abstract

Type 2 diabetes mellitus (T2DM) is a progressive disease. We utilized bioinformatics analysis and experimental research to identify biomarkers indicative of the progression of T2DM, aiming for early detection of the disease and timely clinical intervention. Integrating Mfuzz analysis with differential expression analysis, we identified 76 genes associated with the progression of T2DM, which were primarily enriched in signaling pathways such as apoptosis, p53 signaling, and necroptosis. Subsequently, using various analytical methods, including machine learning, we further narrowed down the hub genes to STK17A and CCT5. Based on the hub genes, we calculated the risk score for samples and interestingly found that the score correlated with multiple programmed cell death (PCD) pathways. Animal experiments revealed that the diabetes model exhibited higher levels of MDA and LDH, with lower expression of SOD, accompanied by islet cell apoptosis. In conclusion, our study suggests that during the progression of diabetes, STK17A and CCT5 may contribute to the advancement of the disease by regulating oxidative stress, programmed cell death pathways, and critical signaling pathways such as p53 and MAPK, thereby promoting the death of islet cells. This provides substantial evidence in support of further disease prevention and treatment strategies.

## Introduction

Diabetes mellitus is a disorder characterized by metabolic anomalies marked by insulin resistance, relative insulin deficiency, and persistent hyperglycemia. The rise in its incidence is propelled by factors such as obesity, nocturnal lifestyle habits, prolonged periods of inactivity, and other detrimental lifestyle choices. Statistically, in 2021, around 537 million adults globally had diabetes, with over 90% diagnosed with type 2 diabetes mellitus (T2DM), leading to roughly 6.7 million deaths worldwide due to the disease and its complications^1^. The microvascular and macrovascular complications arising from T2DM cause significant physical and psychological distress, profoundly impacting global health and economic stability. Despite the widespread recognition of several risk factors for type 2 diabetes and the commercial availability of various antidiabetic drugs, the disease's prevalence remains disturbingly high^2^. Therefore, it is critical to thoroughly investigate the pathogenic mechanisms of T2DM and implement measures that could slow or potentially reverse its course. Precision medicine, a pivotal concept across multidisciplinary fields, aims to unravel the mechanisms that drive the onset and progression of diseases on genetic, transcriptional, and translational levels. In recent decades, integrated analyses of transcriptomic data from specific databases have proven efficacious in identifying new biomarkers and elucidating their biological functions within pathological processes, ushering in new perspectives for understanding disease mechanisms and drug discovery^3^. Nevertheless, in their attempts to forecast T2DM-related biomarkers, researchers like Cui et al.^4^ and Hu et al.^5^ fell short of identifying biomarkers that significantly impact disease progression.

**Figure 1:**
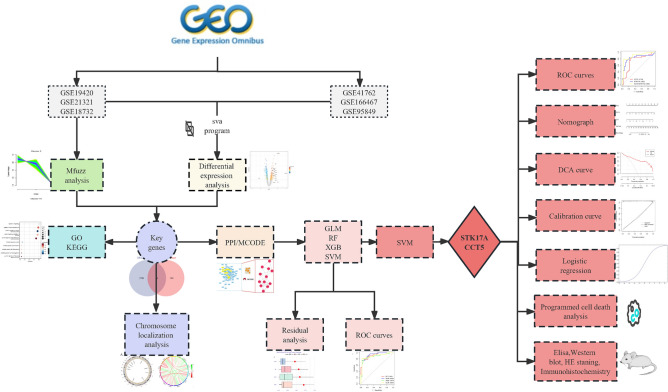
Flowchart of the research. We used bioinformatics analysis and experiments to explore the pathogenesis of type 2 diabetes. (GO/KEGG: gene ontology/Kyoto encyclopedia of genes and genomes; PPI/MCODE: protein–protein interaction/molecular complex detection; generalized linear model (GLM), random forest (RF), extreme gradient boosting (XGB), support vector machine(SVM); receiver operating characteristic curve (ROC); decision curve analysis (DCA); Elisa enzyme-linked immunosorbent assay (Elisa); Hematoxylin–eosin staining (HE staning)).

The World Health Organization (WHO) recommends categorizing T2DM into two pathological states: impaired glucose tolerance (IGT) and T2DM itself, thus conceptualizing T2DM as a continuum that evolves from a normal state to IGT and subsequently to full-blown T2DM^6^. Concentrating research on the onset of T2DM does not facilitate a systematic evaluation of the disease's progression nor promote early intervention. By contrast, the soft clustering algorithm provided by Mfuzz^7^ is adept at capturing the continuous variations within gene expression data. Compared to traditional hard clustering methods, Mfuzz excels in identifying gene similarities and their dynamic changes across diverse biological processes. In our study, we posit the existence of a set of genes that mediate the disease’s transition from a normal state through IGT to T2DM, exhibiting synchronous variations with the disease states. Therefore, this research integrates differential expression analysis, Mfuzz soft clustering, and machine learning algorithms to screen for genetic markers that could indicate the onset and progression of T2DM. Timely identification and intervention targeting these biomarkers could effectively prevent the occurrence of T2DM. We created a flowchart to elucidate our research process (Fig. 1).

## Materials and methods

### Data preparation

Datasets GSE19420, GSE21321, GSE18732, GSE41762, GSE166467, and GSE95849 are accessible through the Gene Expression Omnibus (GEO) database (https://www.ncbi.nlm.nih.gov/geo/). The GSE19420 dataset, which utilizes the GPL570 platform, encompasses 42 samples comprising 12 normal, 12 IGT, 10 T2DM, and 8 T2DM samples post-exercise intervention. The latter 8 samples were omitted from our analysis. On the GPL6883 platform, the GSE21321 dataset contains 24 samples, including 8 normal, 7 IGT, and 9 T2DM. The GSE18732 dataset is based on the GPL9486 platform and includes 118 samples: 47 normal, 26 IGT, and 45 T2DM. Additionally, the GSE95849 dataset, using the GPL22448 platform, consists of 18 samples with 6 normal, 6 T2DM, and 6 diabetic peripheral neuropathy samples; the diabetic peripheral neuropathy samples were excluded from the analysis. Mfuzz analysis was applied to datasets (GSE19420, GSE21321, and GSE18732) containing normal, IGT, and T2DM samples. In contrast, datasets GSE41762, GSE166467, and GSE95849, including normal and T2DM samples, were utilized for logistic regression analysis and subsequent evaluations.

### Mfuzz analysis

In this study, we extracted datasets GSE19420, GSE21321, and GSE18732, and employed the Mfuzz package to cluster gene expression patterns of the aforementioned datasets based on the ordered feature "normal-IGT-T2DM". Subsequently, we extracted clusters of genes that exhibited synchronous changes in expression patterns throughout the "normal-IGT-T2DM" progression. These genes are considered to be strongly associated with the progression of T2DM and were extracted for further analysis.

### Differential expression analysis

We excluded IGT samples from datasets GSE19420, GSE21321, and GSE18732 and then corrected for batch effects in the expression data of six datasets (GSE19420, GSE21321, GSE18732, GSE41762, GSE166467, and GSE95849) using the 'sva' package^8^. To correct for potential batch effects from each dataset, we used the dataset from which each sample originated as the batch source. After merging these datasets into a single dataset, we removed batch effects using the ComBat function from the Sva package. The resulting normalized dataset was then used for subsequent differential expression analysis. To filter for differentially expressed genes, we used the 'limma' package^9^, adopting the criterion of an absolute log2 fold change (|log2FC|≥ 1) and a P-value < 0.05.

### Enrichment analysis

Following the Mfuzz analysis, we discerned specific clusters of genes whose alterations occurred in concert with the evolution of T2DM. Concurrently, we distinguished DEGs. By constructing a Venn diagram, we were able to identify genes that intersected between these clusters and DEGs, regarding these as key genes closely associated with the T2DM. For a comprehensive examination of these essential genes, we conducted Gene Ontology (GO) and Kyoto Encyclopedia of Genes and Genomes (KEGG) enrichment analyses^10–12^ via the Sangerbox tool^13^. The results of the enrichment analysis were refined employing an adjusted P-value cutoff of less than 0.05.

### Protein–protein interaction (PPI) network analysis

The identified key genes were uploaded to the STRING database^14^, with the species specified as Homo sapiens and the minimum required interaction score set to 0.150. This facilitated the construction of a protein–protein interaction (PPI) network, which was subsequently imported into Cytoscape software version 3.7.1^15^ for network topology analysis. Within Cytoscape, the Molecular Complex Detection (MCODE) algorithm was employed to discern functional clusters of genes within the PPI network. The parameters for the MCODE analysis included a degree cutoff of 2, a node score cutoff of 0.2, a k-score of 2, and a maximum depth of 100^16^. The highest-scoring cluster identified by MCODE was selected, and the genes within this cluster were extracted for additional investigation.

### Construction, evaluation, and forecasting of predictive models

The dataset underwent random partitioning to form a training subset comprising 70% of the original data and a testing subset containing 30%. Key genes were utilized as predictive attributes, with cases of T2DM designated as 1 to indicate the positive class and non-disease samples marked as 0 to signify the harmful category. Within the R computational environment, four prevalent classifier algorithms were developed utilizing the randomForest^17^, xgboost^18^, and caret^19^ packages: Generalized linear model (GLM), random forest (RF), extreme gradient boosting (XGB), and support vector machine (SVM). The interpretability of the machine learning algorithms was enhanced using the DALEX package, which facilitated the assessment of feature significance through Permutation Feature Importance (PFI) and the computation of model residuals. The efficacy of the classifiers was quantified by executing tenfold cross-validation employing the pROC package, which involved the generation of Receiver Operating Characteristic (ROC) curves and the determination of area under the curve (AUC) metrics. Clinical decision curves, calibration plots, and nomograms were also constructed using the rms package^20^ to evaluate the models' clinical applicability further.

The selection of the superior model rested upon attaining the minimal residuals and the maximal AUC. The relative significance of the predictor variables was quantified, identifying two hub genes. A prognostic model for these hub genes was formulated using the top-performing classifier and subsequently verified against the testing subset. ROC curves were plotted to ascertain the predictive model's accuracy. The model's clinical utility was examined via decision curve analysis, while its predictive precision was checked through calibration plots. Lastly, a nomogram was devised to represent the model's predictive power visually.

### Risk scores and programmed cell death(PCD)

A multifactorial logistic regression model was constructed employing hub genes as covariates. For each hub gene, odds ratio (OR) was computed, and risk scores were derived for the samples correlating to their expression levels. The samples were stratified into high-risk and low-risk cohorts predicated on their risk scores median value.

Thirteen gene sets pertinent to programmed cell death (PCD) were curated from extant literature and various databases^21–33^, and the GSVA^34^ package was utilized to compute PCD scores for the samples. The correlation function was employed to estimate the Spearman correlation coefficients, elucidating the relationship between PCD scores and risk scores.

### Animals

Permission for this study was obtained from the Laboratory Animal Ethics Committee of Kangtai Medical Laboratory Service Hebei Co., Ltd. (MDL2023-06-28-03). All methods were performed in accordance with the relevant guidelines and regulations. Six 8-week-old male SD rats purchased from the Animal Experiment Center were randomly divided into T2DM group (3 rats) and control group (3 rats). For eight weeks, rats in the T2DM group received a high-fat diet (HFD; 60% fat), while rats in the control group had a normal diet (NCD). The average lab temperature was 25 °C, and there was a 12-h light/dark cycle. Subsequently, the T2DM group of rats were then intraperitoneally administered 30 mg/kg of streptozotocin (STZ) dissolved in 0.1 M sterile citrate buffer (pH = 4.5) for 1 weeks. The control rats were injected with the same dose of sodium citrate solution. We deemed the rat modeling successful when the blood glucose level in the caudal capillaries was more than 11.1 mmol for more than 3 successive readings. Extract blood from the posterior orbital vein of rats for subsequent analysis. After euthanizing the rats by inhaling an overdose of isoflurane, we removed the islets and weighed them.

### Islet function testing

The levels of insulin (TZGJEY12XE, Elabscience Biotechnology Co., Ltd, Wuhan, China) and glycosylated serum protein (GSP) (FY-A014679, Shanghai Jianglai Biotechnology Co., Ltd, Shanghai, China) in rats were measured separately according to the instructions of the elisa kit.

### Examination of oxidative stress

Lactate dehydrogenase (LDH) (BLL-hlk3939), malondialdehyde (MDA) (BLL-yx3496) and superoxide dismutase (SOD) (BLL-yx3497) assay kits were purchased from Shanghai Jianglai Biotechnology Co., Ltd, Shanghai, China. We performed ELISA assays on frozen samples to assess the level of oxidative stress (OS) according to the kit instructions.

### Western blot

Rats’ islet tissue was lysed in RIPA lysis buffer containing protease inhibitors, then centrifuged and the supernatant was collected. After separating the proteins in the samples using 10% PAGE electrophoresis, the samples were transferred to PVDF membranes. The membranes were closed with TBST containing 5% skim milk powder for 2 h and incubated with primary antibody and secondary antibody in that order. GAPDH was the internal reference proteins of CCT5 and STK17A, respectively. Finally, protein strips were detected using ProteinSimple, and grayscale analysis was performed using ImageJ software. Antibodies were provided by Abcam Trading (Shanghai) Co., Ltd, including Anti-STK17A antibody (ab8418, abcam), Anti-TCP1 epsilon/CCT5 antibody [EPR7562] (ab129016, abcam), Anti-GAPDH antibody [6C5]—Loading Control (ab8245, abcam).

### Pathological changes in pancreatic islets

The 4% paraformaldehyde-fixed pancreatic tissues were routinely paraffin-embedded, sectioned and stained with HE, and the pathological changes of pancreatic islets were observed under the light microscope at 100x.The key part of the pictures were enlarged to 4 times.

### Expression of apoptosis biomarkers by immunohistochemistry

Paraffin sections of pancreatic tissue were taken, dewaxed and hydrated, antigen repaired, and endogenous peroxidase activity eliminated and closed. Anti-Bax antibody (ab32503, Abcam), Anti-Bcl-2 antibody (ab182858, Abcam), and Anti-Caspase-3 antibody (ab32351, Abcam) from Abcam Trading (Shanghai) Co., Ltd were incubated at 37 ℃ for 60–120 min, secondary antibodies were set at 37 ℃ for 0.5–2 h, and DAB was used for colour development. Hematoxylin re-staining, dehydration, transparency and sealing were performed and photographed at 100x. The main part of the pictures were enlarged to 4 times. The percentage of positive area for Bax, Bcl- 2 and Caspase-3 was analyzed. Bax and Bcl-2 were expressed in the cell plasma, and Caspase-3 was expressed in both the cell plasma and nucleus and appeared as brown or tan particles.

### Statistical analysis

We conducted the statistical analysis using R 4.1.3 software. Normally distributed measures were expressed as mean ± standard deviation, denoted as x ± s. In case of unequal variances, the Wilcox test was utilized. Statistical significance was determined at a threshold of P < 0.05.

## Results

### Mfuzz analysis

**Figure 2:**
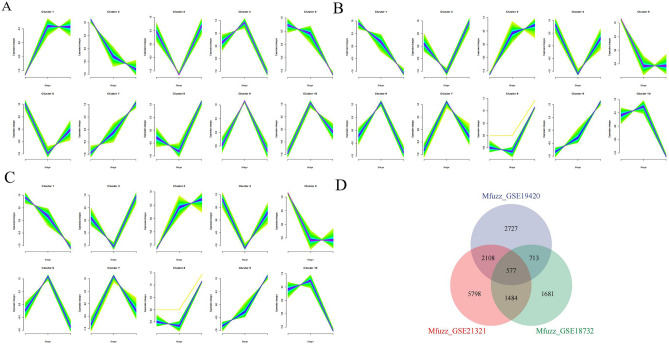
Mfuzz analysis. (**A**) GSE19420 (**B**) GSE21321 (**C**) GSE18732 (**D**) Venn diagram of the Mfuzz results.

In the GSE18732 dataset, cluster 7 exhibited synchronous upregulation, while cluster 2 and cluster 5 showed downregulation (Fig. 2A). In the GSE19420 dataset, clusters 3 and 9 demonstrated upregulation, while Cluster 1 displayed downregulation (Fig. 2B). In the GSE21321 dataset, clusters 1 and 7 showed upregulation, whereas Clusters 3 and 6 showed downregulation (Fig. 2C). We extracted the genes within these clusters, resulting in 577 intersecting genes according to the Venn diagram (Fig. 2D).

### Differential expression analysis

**Figure 3:**
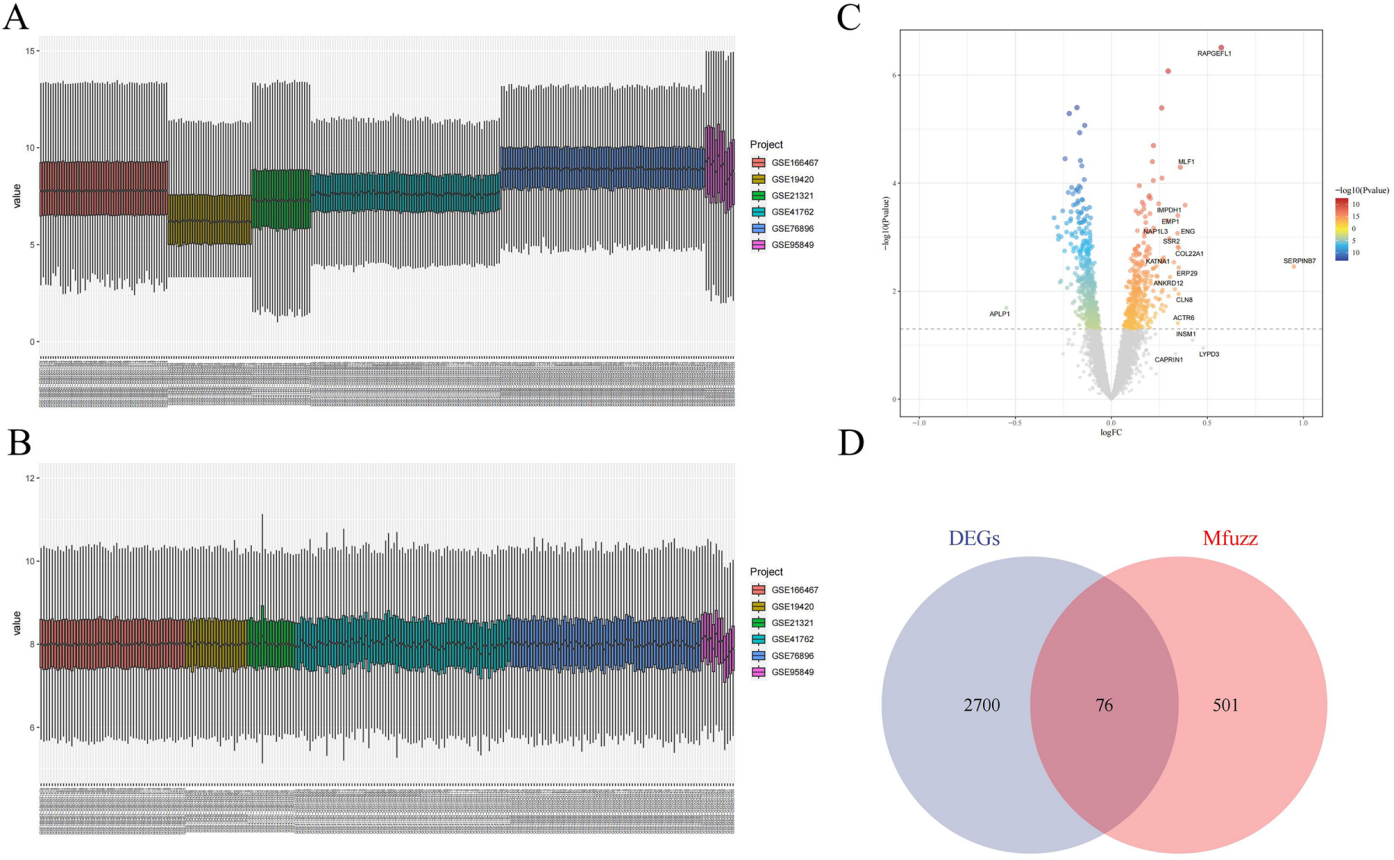
Differential expression analysis. (**A**) Box plot of gene expression before removal of batch effects (**B**). Box plot of gene expression after removal of batch effects (**C**) Volcano plot (**D**) Venn diagram of DEGs and Mfuzz results.

Six datasets (GSE19420, GSE21321, GSE18732, GSE41762, GSE166467, and GSE95849) exhibited batch effects before merging (Fig. 3A). After removing the batch effects using the sva utility, the gene expression box plots of the datasets showed nearly identical shapes (Fig. 3B), indicating the successful removal of batch effects. The merged datasets were then subject to differential analysis using the limma package, identifying 2776 differentially expressed genes (DEGs) at a significance level of |logFC|≥ 1 & P < 0.05. Among them, 1257 genes were up-regulated, and 1519 were down-regulated (Fig. 3C). We presented the overlapping genes between the differential and Mfuzz analyses in a Venn diagram, identifying 76 intersecting genes as key genes for T2DM development (Fig. 3D).

### GO and KEGG enrichment analysis

**Figure 4:**
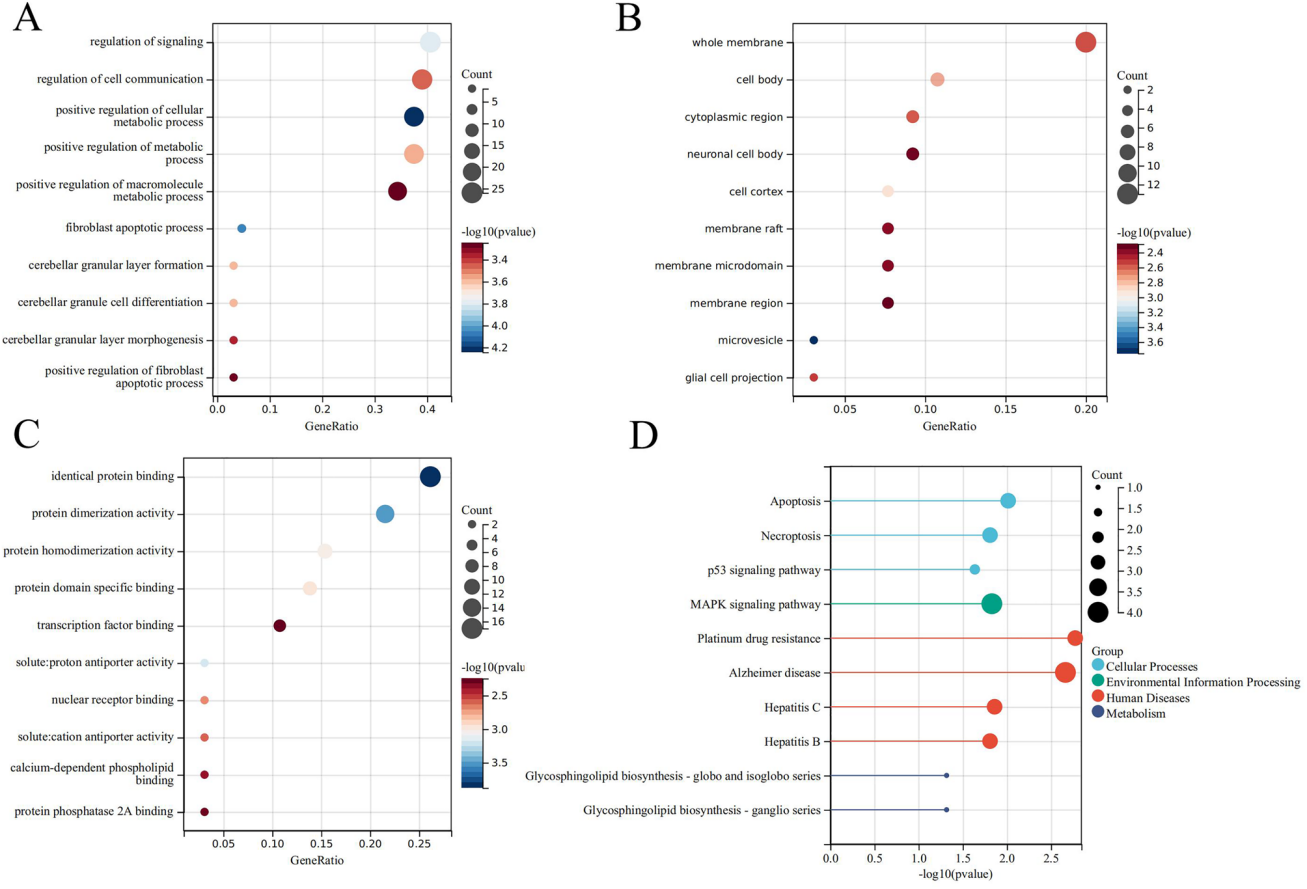
Enrichment analysis. (**A**) BP (**B**) CC (**C**) MF (**D**) KEGG.

Key genes are primarily enriched in the following biological processes (BP): regulation of signalling, apoptosis, metabolic process positive, regulation of the metabolic process, etc. (Fig. 4A). Key genes are primarily enriched in the following cellular components (CC): whole membrane, cell body, etc. (Fig. 4B). And molecular functions (MF) are primarily enriched in identical protein binding, protein dimerization activity, protein homodimerization, etc. (Fig. 4C). T2DM-related essential genes were largely abundant in the p53 signalling pathway, MAPK signalling pathway, apoptosis and necroptosis pathways, according to KEGG enrichment analysis (Fig. 4D).

### Protein–protein interaction network analysis

**Figure 5:**
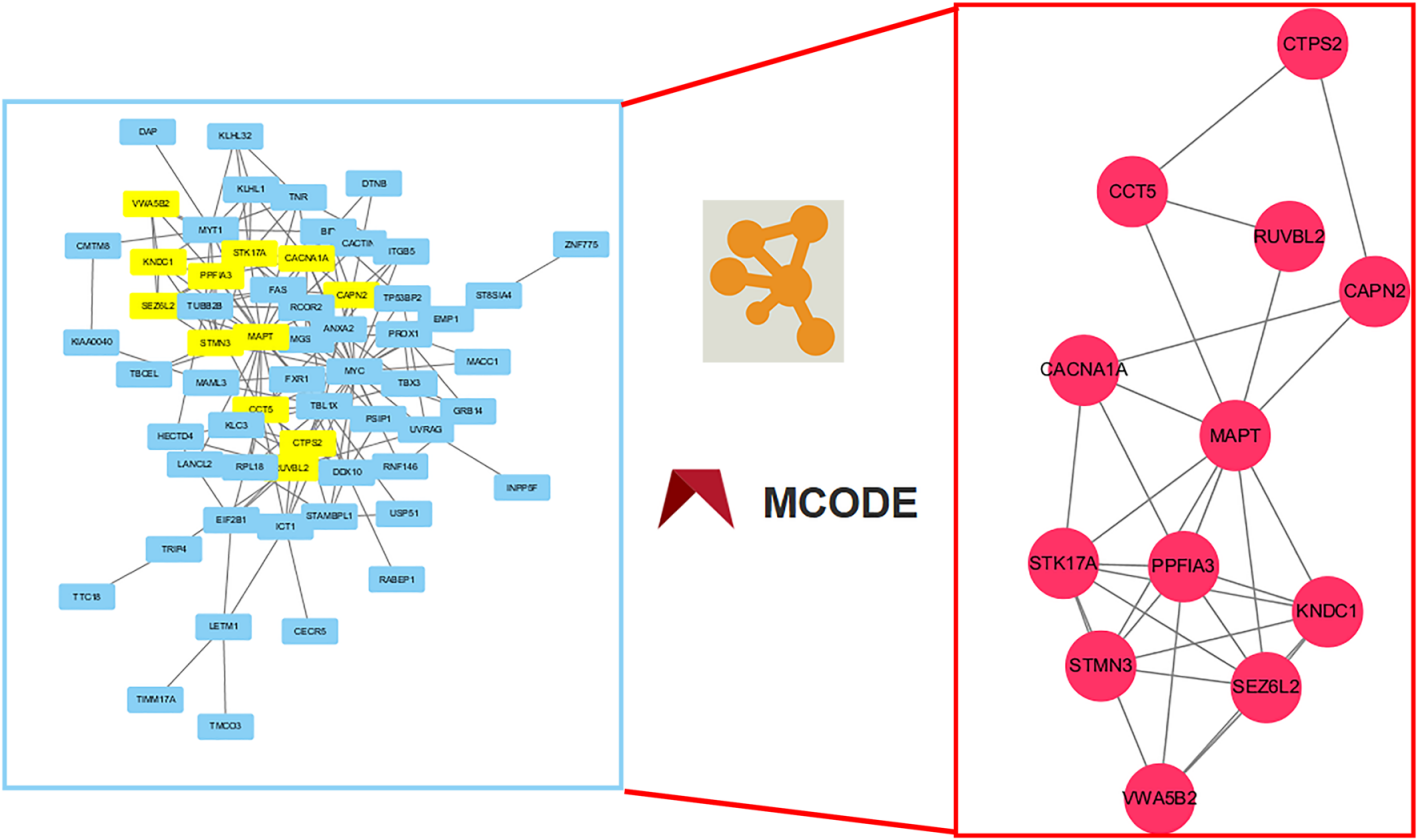
PPI network. PPI network (left side) and the highest scoring subnetwork recognized by the MCODE plugin (right side).

The PPI network was constructed by inputting key genes into the STRING database. After eliminating isolated nodes, the network comprised 74 nodes and 174 links or edges. The network was then imported into Cytoscape 3.7.1 software for network topology analysis. Utilizing the MCODE plug-in, the network was partitioned into three clusters. Cluster 1 comprised 12 nodes (CCT5, STMN3, STK17A, RUVBL2, KNDC1, CACNA1A, CTPS2, PPFIA3, MAPT, SEZ6L2, CAPN2, VWA5B2) with 29 edges and a score of 5.273. Cluster 2 consisted of nine nodes (TNR, TP53BP2, TUBB2B, BID, FAS, KLHL32, RCOR2, MYT1, KLHL1) with 14 edges and a score of 3.5. Cluster 3 included three nodes (DDX10, FXR1, PSIP1) with three edges and a score of 3 (Fig. 5).

### Key gene chromosomal localization and correlation

**Figure 6:**
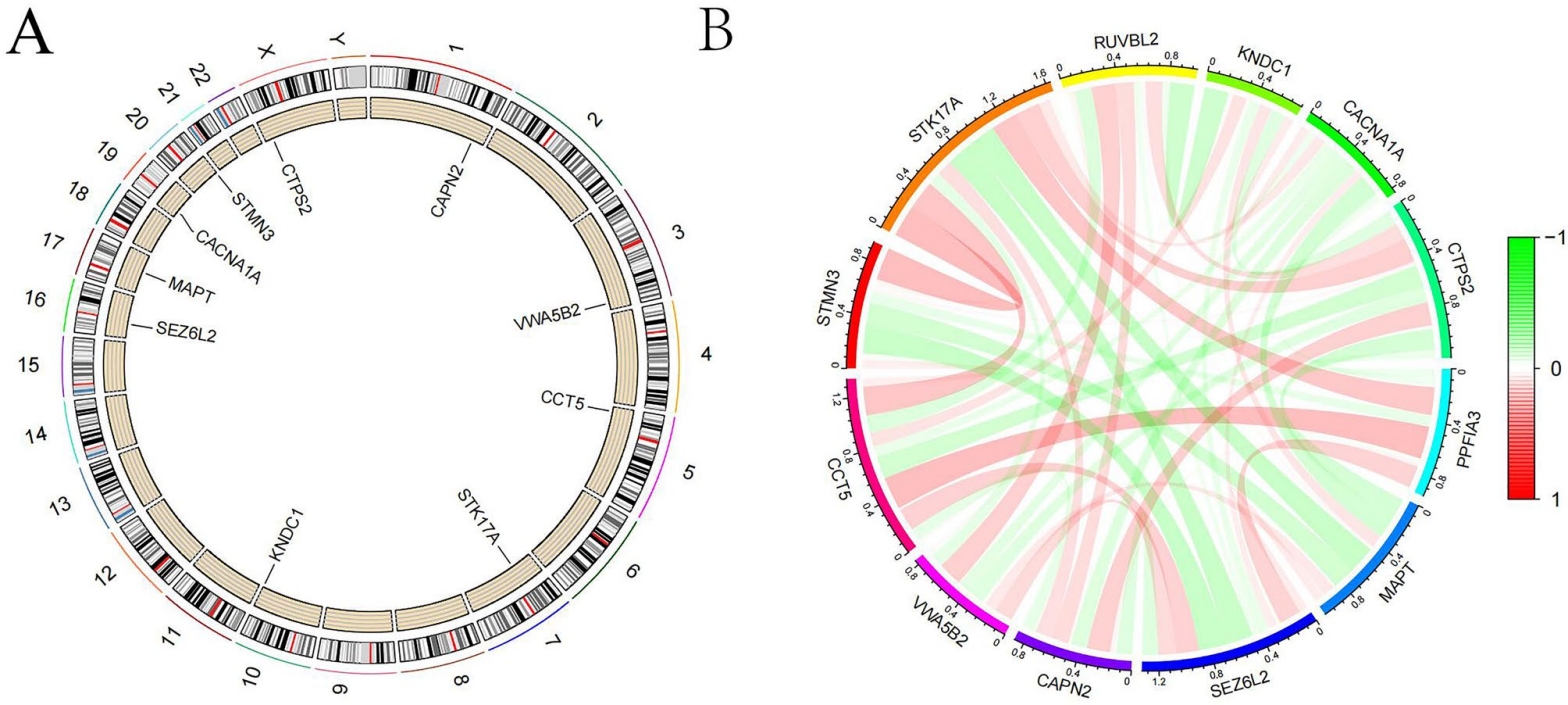
Chromosome localization and correlation analysis. (**A**) Chromosomal localization of key genes (**B**) Correlation chord diagram of key genes.

Except for CTPS2, all key genes were found on autosomes (Fig. 6A), and they were all related to one another (Fig. 6B).

### Construction, evaluation, and forecasting of predictive models

**Figure 7:**
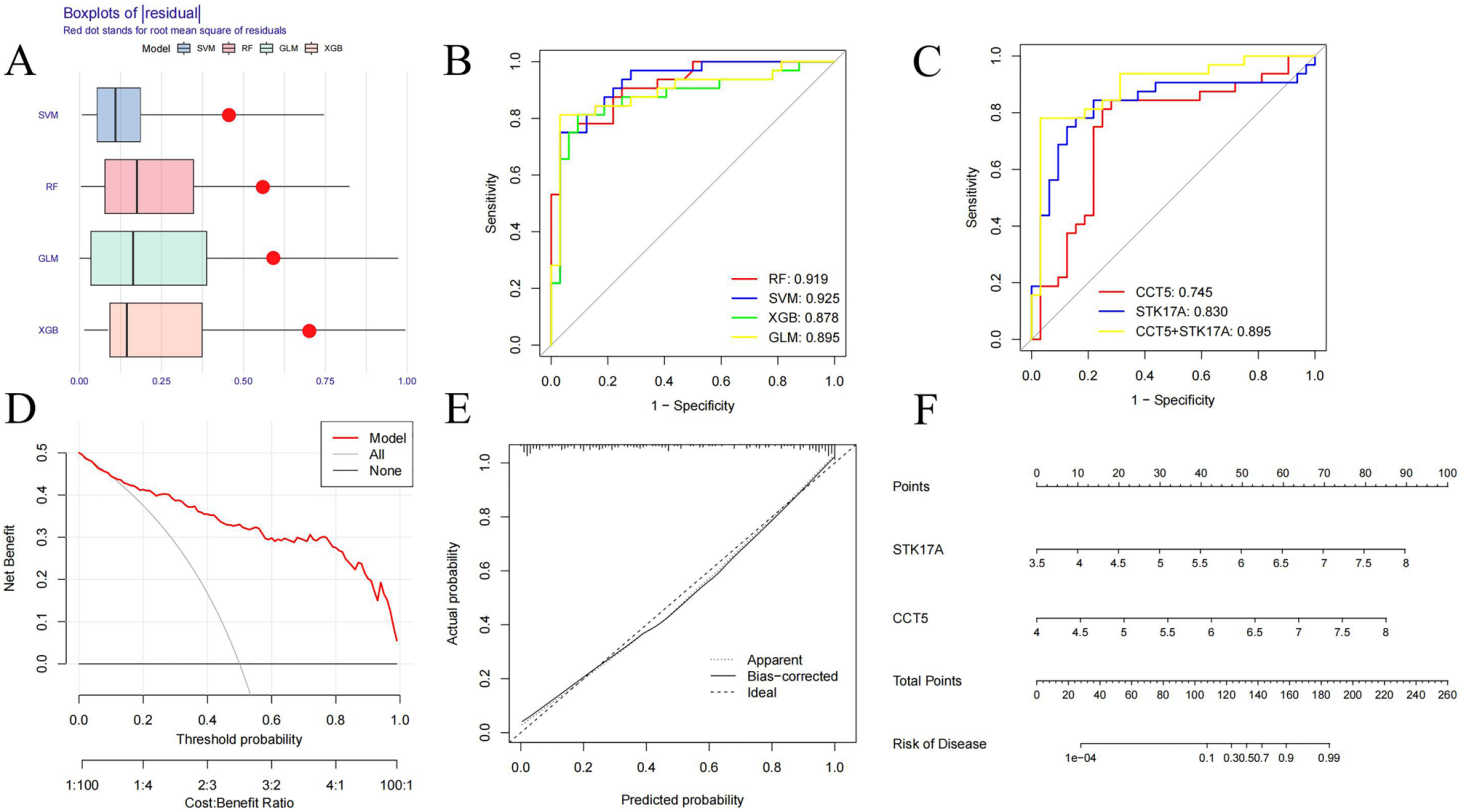
Machine learning models. (**A**) Residual accumulation of different classifiers (**B**). ROC curves of different classifier models (**C**). ROC curves of SVM models based on hub genes (**D**) DCA curves (**E**) Calibration curves (**F**) Nomograms.

Based on the model, we plotted nomogram for visualization (Fig. 7F).We developed prediction models using SVM, RF, GLM, and XGB on the training set, focusing on the key genes. The SVM model displayed the highest prediction performance with an AUC of 0.925 (Fig. 7B) and lower residuals (Fig. 7A), making it the optimal model. We calculated the relative importance of the feature variables in the SVM model using the DALEX package, which identified CCT5 and STK17A as the two most significant hub genes. Then, we constructed an SVM prediction model using these hub genes on the test set, which showed promising prediction performance on the test set with an AUC of 0.895 (Fig. 7C). Furthermore, the clinical decision curve analysis (Fig. 7D) demonstrated the net benefit of the hub genes. In contrast, the calibration curve results (Fig. 7E) indicated that the SVM model's prediction probabilities based on hub genes aligned closely with the true probabilities. To visualize the model, we created a nomogram (Fig. 7F).

### Risk scores and PCD correlation

**Figure 8:**
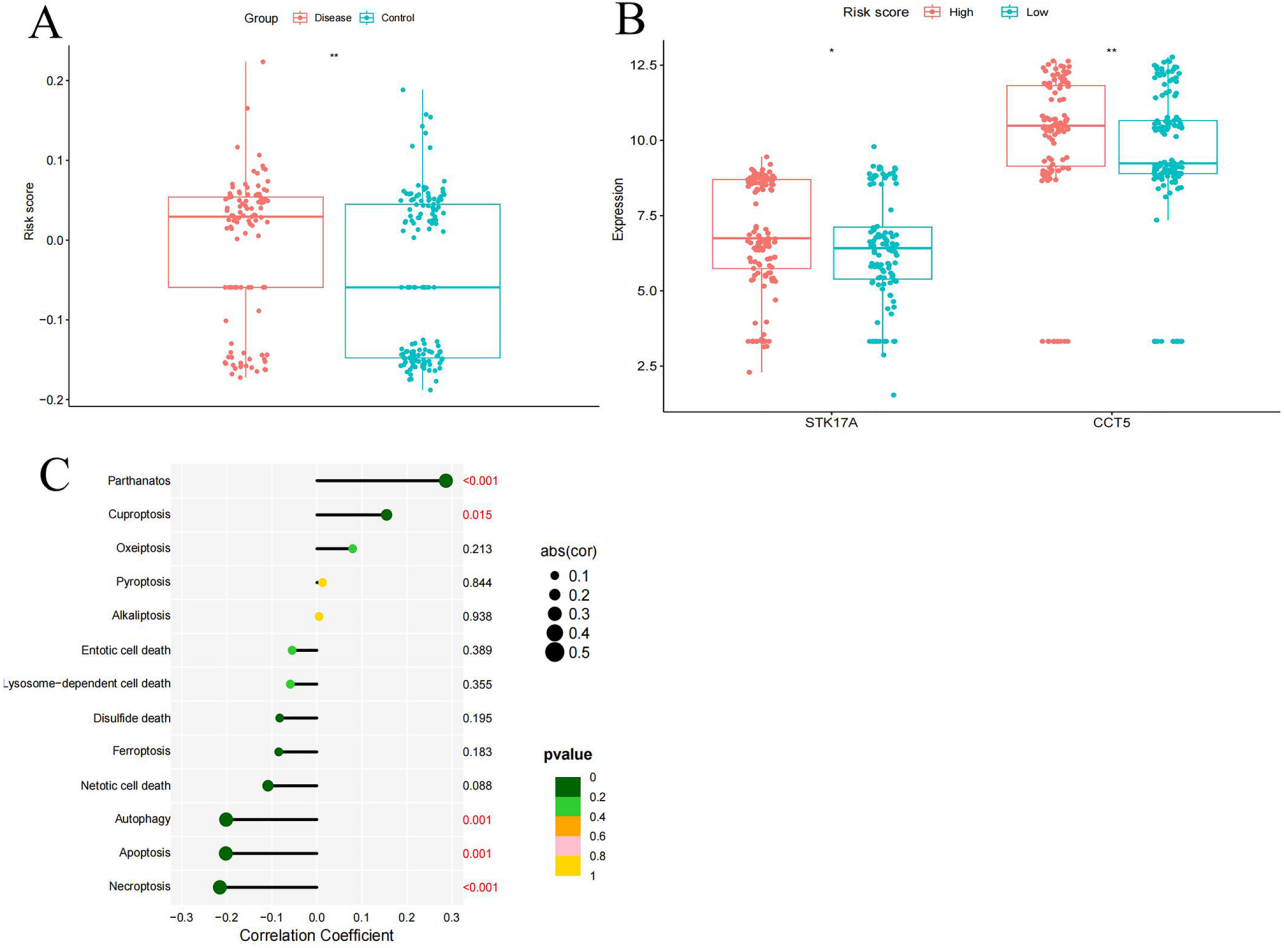
Risk score comparison. (**A**) RS in T2DM vs control group (**B**) hub genes in high RS vs low RS group (**C**) RS vs PCD correlation.

The sample risk score (RS) was calculated using RS = 1.05 × STK17A + 1.03 × CCT5. We observed that the T2DM group exhibited a significantly higher RS than other groups (P < 0.05) (Fig. 8A). Additionally, the high RS group demonstrated elevated expression levels of both STK17A and CCT5 (Fig. 8B). Among the 13 PCDs investigated, the RS showed significant associations with Parthanatos, Cuproptosis, Autophagy, Apoptosis, and Necroptosis (P < 0.05) (Fig. 8C).

### Comparison of ELISA results

**Figure 9:**
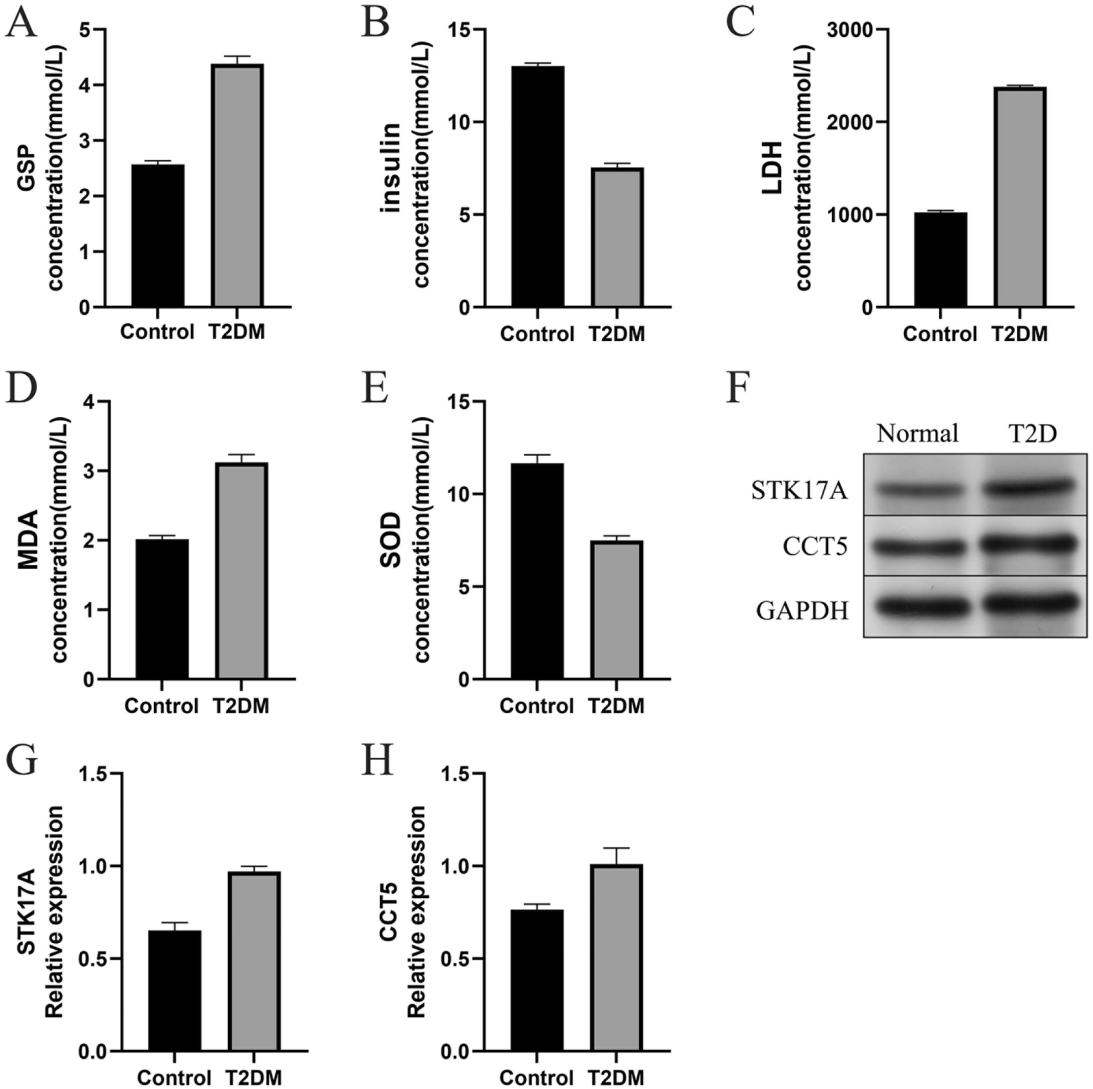
Result of Elisa and western blot. (**A**–**E**) Elisa result for GSP, insulin, LDH, MDA and SOD (**F**) western blot result of hub genes (**G**,**H**) Grayscale analysis of STK17A and CCT5.

Compared to the control group, rats in the T2DM group had increased levels of GSP (Fig. 9A) and decreased levels of fasting insulin (Fig. 9B). This indicates that islet function was significantly reduced in the T2DM group of rats. Meanwhile, LDH (Fig. 9C) and MDA (Fig. 9D) levels were significantly increased, while SOD (Fig. 9E) activity was significantly decreased in the rats of the T2DM group.This suggests that rats in the T2DM group had increased levels of oxidative stress and decreased antioxidant capacity.

### Western blot results

After Western blot and grayscale analysis, we found the same expression trend as the above results, with higher expression levels of key genes, namely STK17A, and CCT5 proteins, in T2DM rats compared to controls (Fig. 9F–H). Note that Fig. 9F is a cropped image of the westernblot of the original gel presented in Supplementary Material.

### Comparison of the pathological morphology of islets

**Figure 10:**
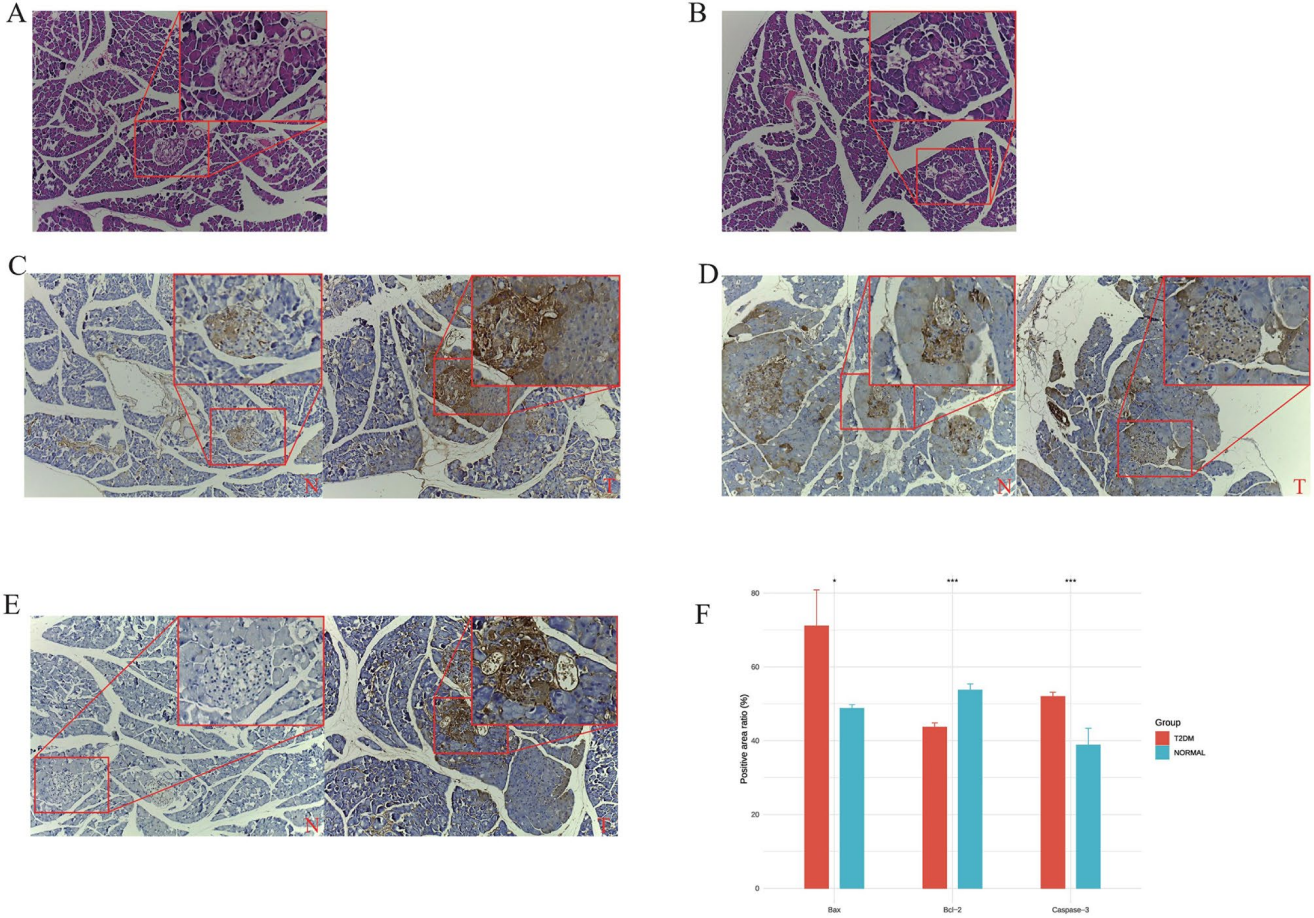
HE staining and IHC results. (**A**,**B**) HE staining pancreas of normal pancreas and T2DM sample (**C**–**E**) Bax , Bcl-2 and Caspase-3 immunohistochemical results of normal pancreas (left) and T2DM pancreas (right) (**F**) Comparison of Bax, Bcl-2 and Caspase-3 positive area percentage of pancreas in each group.

Rats in the control group had clear islet cell structures and high numbers (Fig. 10A). In contrast, rats in the T2DM group had significantly damaged islet cell structure and significantly reduced numbers (Fig. 10B). Compared with T2DM rats, the percentage of pancreatic Bax (Fig. 10C) and Caspase-3 (Fig. 10D) positive area was decreased (P < 0.05) (Fig. 10F) and the percentage of Bcl-2 (Fig. 10E) positive area was increased (P < 0.01) in control rats (Fig. 10F).

## Discussion

This study utilized bioinformatics analysis and experimental research to identify biomarkers indicative of the progression of T2DM, aiming for early disease detection and timely clinical intervention. Integrating Mfuzz analysis with differential expression analysis, we identified 76 genes associated with the progression of T2DM. KEGG pathway enrichment analysis revealed that the 76 key genes identified exhibited significant enrichment in various pathways, most notably in apoptosis, p53 signaling, MAPK signaling, and necroptosis. Utilizing an array of analytical approaches, including PPI networks, MCODE, and SVM analysis, we identified two central hub genes: STK17A and CCT5. The risk score, formulated based on these hub genes, displayed correlations with numerous PCD pathways. Moreover, in vivo experiments using SD rats confirmed the gene expression patterns of these hub genes, aligning with results from differential expression analysis. Significantly, the rats demonstrated elevated levels of MDA and LDH, alongside a reduction in SOD activity and increased apoptosis within islet cells. These observations imply that STK17A and CCT5 may play pivotal roles in the pathogenesis and evolution of IGT and T2DM. They appear to exert their influence by modulating pathways associated with oxidative stress, programmed cell death, and critical signal transduction pathways such as p53 and MAPK, ultimately contributing to islet cell apoptosis.

STK17A, or death-associated protein kinase-related apoptosis-inducing protein kinase 1 (DRAK1), is a member of the death-associated protein kinase (DAPK) family and is known to positively regulate apoptosis^35,36^. This kinase has been identified as pivotal in many cellular processes, such as cell proliferation, apoptosis, tumor metastasis, and tumorigenesis^37,38^. Although the involvement of STK17A in cancer is well-documented, recent research has also highlighted its importance in non-cancerous diseases. For example, Li et al. found that miR-182-5p targets STK17A results in an elevated apoptosis rate and increased levels of ROS^39^. CCND1, a recognized cell cycle regulator with links to tumorigenesis and proliferation^40^, has also been implicated in the migration and invasion of tumor cells. Within its network of interacting proteins, the chaperonin containing TCP1 subunit 5 (CCT5) has been shown to play a crucial role^41^. Studies have indicated that CCT5 has a high affinity for ATP and can prevent the accumulation of aberrant proteins^42,43^. Remarkably, the expression of CCT5 protein in E. coli has unveiled chaperone activity that was not anticipated^44^. Additionally, the suppression of the CCT5 gene modified the responsiveness of small-cell lung cancer to chemotherapy^45^, and an increase in CCT5 expression has been correlated with decreased sensitivity of breast cancer cells to doxorubicin in instances involving p53 mutations^46^. However, the interplay between STK17A, CCT5, and T2DM remains elucidated. In the present study, we have performed comprehensive bioinformatics analyses and animal experiments to propose that STK17A and CCT5 could be potential therapeutic targets for the early intervention of T2DM. Nevertheless, further experimental studies are essential to confirm these findings.

The MAPK (mitogen-activated protein kinase) signalling pathway regulates diverse physiological processes, including cell growth, differentiation, inflammation, and apoptosis. In diabetes research, the MAPK signalling pathway is considered an important pathway closely related to insulin resistance and T2DM development. MAPK signalling pathway comprises ERK, JNK and p38 MAPK, and the ERK kinase pathway plays a key role in insulin signalling. It was discovered that diminished ERK kinase activity might impair insulin signalling, promoting insulin resistance and type 2 diabetes^47^.The JNK kinase and p38 MAPK pathways in cellular stress and inflammatory responses play important functions. Activation of the p38 MAPK pathway was found to be associated with insulin resistance and T2DM development^48^. Wu^49^ discovered that activated natural product flavonoids (such as quercetin) can reduce insulin resistance and inflammation by inhibiting JNK and p38 MAPK signaling pathways, thereby lowering the risk of developing T2DM. Tea polyphenols, an antioxidant found in green tea, can improve insulin sensitivity and anti-inflammatory effects by inhibiting ERK and JNK signaling pathways, thus contributing to the prevention and treatment of T2DM. As a transcription factor, the well-known tumor suppressor protein p53 is essential for controlling the cell cycle, apoptosis, and DNA repair. It has been discovered that p53 has the ability to control IRS1 (insulin receptor substrate 1) and Akt, two important signaling molecules in the insulin signaling pathway. Impairment of insulin signaling brought on by abnormal p53 activation may worsen insulin resistance and T2DM^50^. The capacity for secreting insulin is decreased by islet apoptosis, which is brought on by excessive p53 activity. This has significant effects on the onset of T2DM because low insulin secretion results in high blood sugar levels^51^. Yuan^52^ later discovered that SIRT1 can block its function by deacetylating p53, improving insulin signaling and lowering insulin resistance. The prevention and treatment of T2DM may benefit from SIRT1 activators, such as resveratrol. EGCG (epigallocatechin gallate), a natural substance According to studies, EGCG can stop p53 from functioning, which lowers insulin resistance and enhances pancreatic beta-cell function^53^.

The findings of this study's animal studies led to the conclusion that hub genes may influence T2DM by controlling oxidative stress. The major enzymes that produce free radicals are nicotinamide adenine dinucleotide phosphate (NADPH) oxidase and the mitochondrial respiratory chain (MRC)^54,55^, whereas -cells have the little antioxidant capability. As a result, oxidative stress decreases the activity of beta cells through various pathways, including NF-B, p38 MAPK, and JNK/SAPK. It even interferes with their ability to proliferate and differentiate^56^, as well as causing senescence and apoptosis in them^57,58^. Excessive levels of free radicals can seriously compromise glucose homeostasis and proper insulin signalling. Inhibiting -cell malfunction by antioxidation is a novel and interesting treatment approach for T2DM, as Doaa A^59^ has shown through animal research that melatonin can boost -cell regeneration and safeguard the insulin-producing ability of -cells.

This study aimed to consolidate various algorithms, multiple datasets, and animal experiments to authenticate the credibility of the findings. Nonetheless, the study does have certain inevitable limitations. Further rescue experiments are needed to verify the significance of the key genes identified in this study as vital targets for regulating programmed cell death (PCD) and oxidative stress, ultimately enhancing pancreatic-cell functionality. Conversely, validation through subsequent clinical trials remains necessary.

## Conclusions

In conclusion, this study proposes a potential pathogenetic mechanism underlying the progression from normol to IGT and subsequently to T2DM. It highlights STK17A and CCT5 as potential therapeutic targets for T2DM and establishes a robust predictive model for disease progression. Moreover, the study underscores the importance of PCD and oxidative stress as prospective biomarkers of critical relevance. Nevertheless, further experimental validation is essential to corroborate these findings.

### Supplementary Information

Supplementary Information.

## Data Availability

The datasets generated and/or analysed during the current study are available in the GEO repository (www.ncbi.nlm.nih.gov/geo/). GSE123568 is available at: https://www.ncbi.nlm.nih.gov/geo/query/acc.cgi?acc=GSE19420. GSE21321 is available at: https://www.ncbi.nlm.nih.gov/geo/query/acc.cgi?acc=GSE21321. GSE18732 is available at: https://www.ncbi.nlm.nih.gov/geo/query/acc.cgi?acc=GSE18732. GSE41762 is available at: https://www.ncbi.nlm.nih.gov/geo/query/acc.cgi?acc=GSE41762. GSE166467 is available at: https://www.ncbi.nlm.nih.gov/geo/query/acc.cgi?acc=GSE166467. GSE95849 is available at: https://www.ncbi.nlm.nih.gov/geo/query/acc.cgi?acc=GSE95849.
