# Author Correction: Biomarker screening using integrated bioinformatics for the development of “normal—impaired glucose intolerance—type 2 diabetes mellitus”

**DOI:** 10.1038/s41598-024-59805-x

**Published:** 2024-04-25

**Authors:** Dongqiang Luo, Xiaolu Gao, Xianqiong Zhu, Jiongbo Xu, Pengfei Gao, Jiayi Zou, Qiaoming Fan, Ying Xu, Tian Liu

**Affiliations:** 1 Guangzhou University of Chinese Medicine, Guangzhou, 510000 China; 2 Yunkang School of Medicine and Health, Nanfang College Guangzhou, Guangzhou, 510000 China; 3 Foshan Hospital of Traditional Chinese Medicine, Foshan, 528000 China

Correction to: *Scientific Reports* 10.1038/s41598-024-55199-y, published online 24 February 2024

In the original version of this Article, Tian Liu was incorrectly affiliated with ‘The Fourth School of Clinical Medicine, Guangzhou University of Chinese Medicine, Shenzhen, 518000, China.’. The correct Affiliation is listed below:

Tian Liu:

‘Foshan Hospital of Traditional Chinese Medicine, Foshan, 528000, China.’

The original Article has been corrected.

