# Supplementary material for: Biomarker screening using integrated bioinformatics for the development of “normal—impaired glucose intolerance—type 2 diabetes mellitus”
Source: Sci Rep. 2024 Feb 24;14:4558. doi: 10.1038/s41598-024-55199-y (PMC10894242; doi:10.1038/s41598-024-55199-y)

HE staining

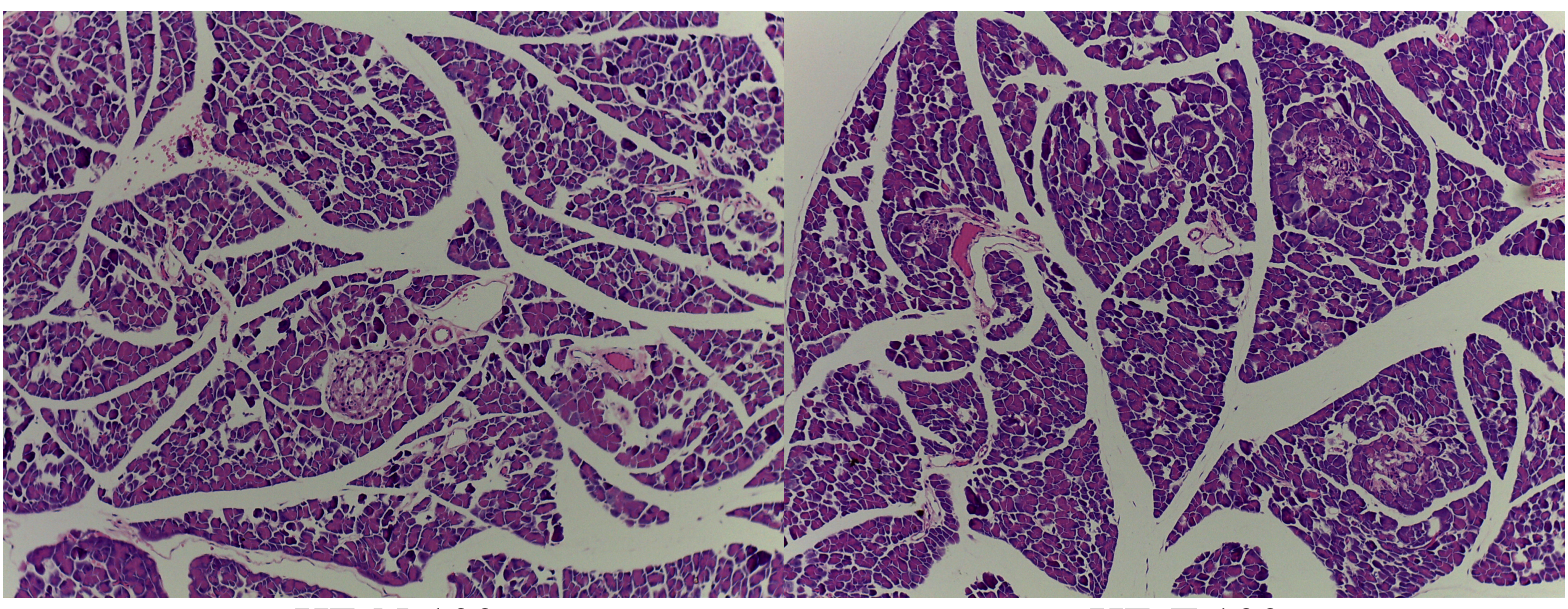

HE-N-100 HE-T-100

Immunohistochemical

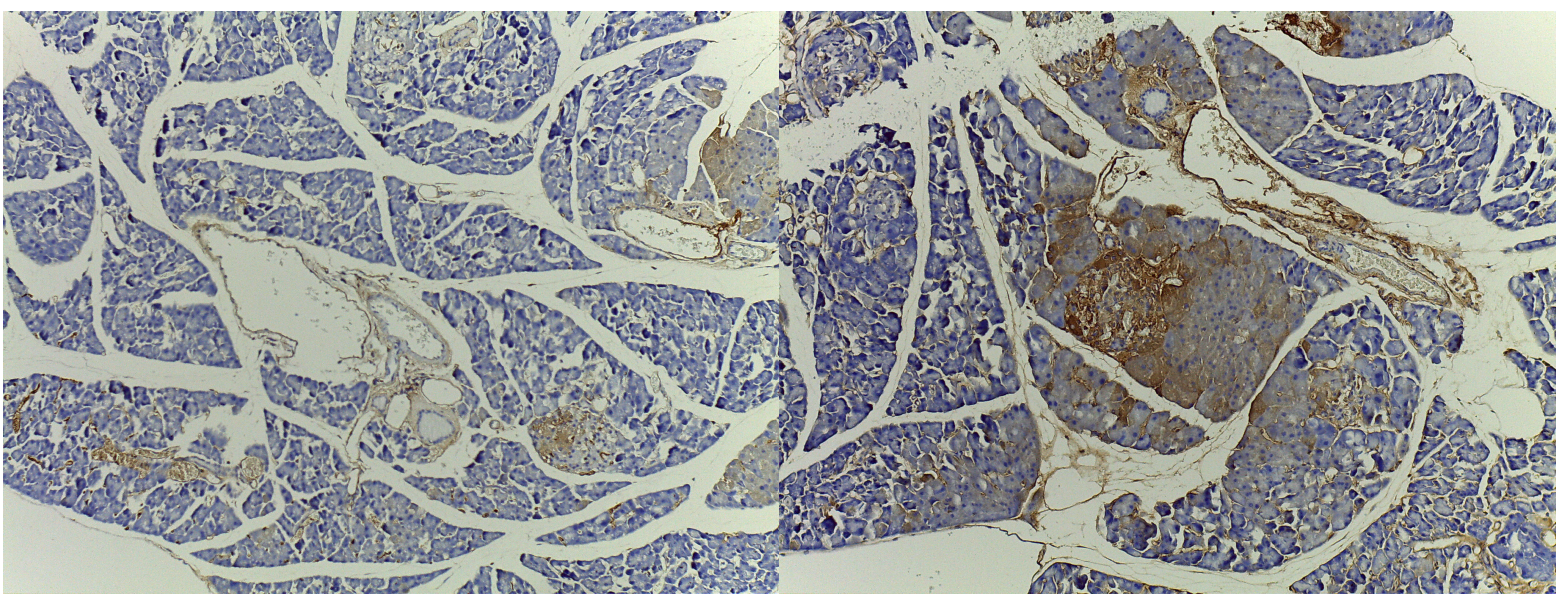

Bax-N-100 Bax-T-100

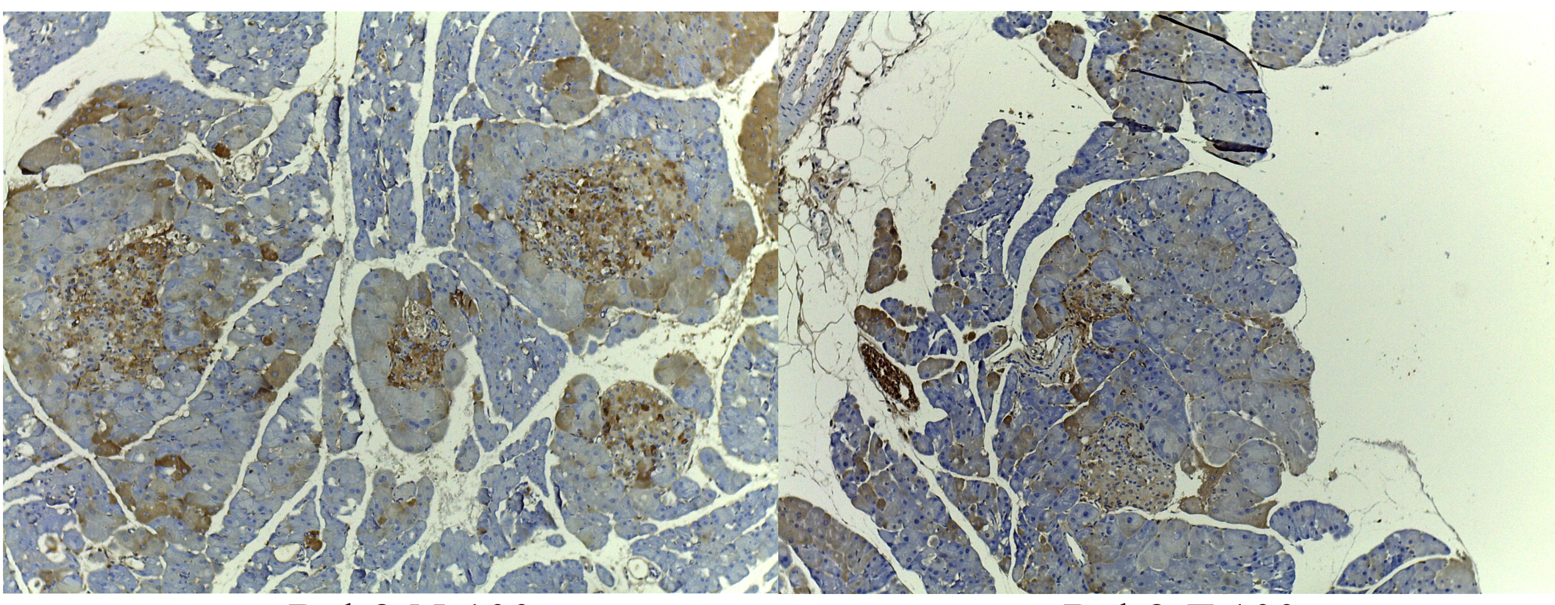

Bcl-2-N-100 Bcl-2-T-100

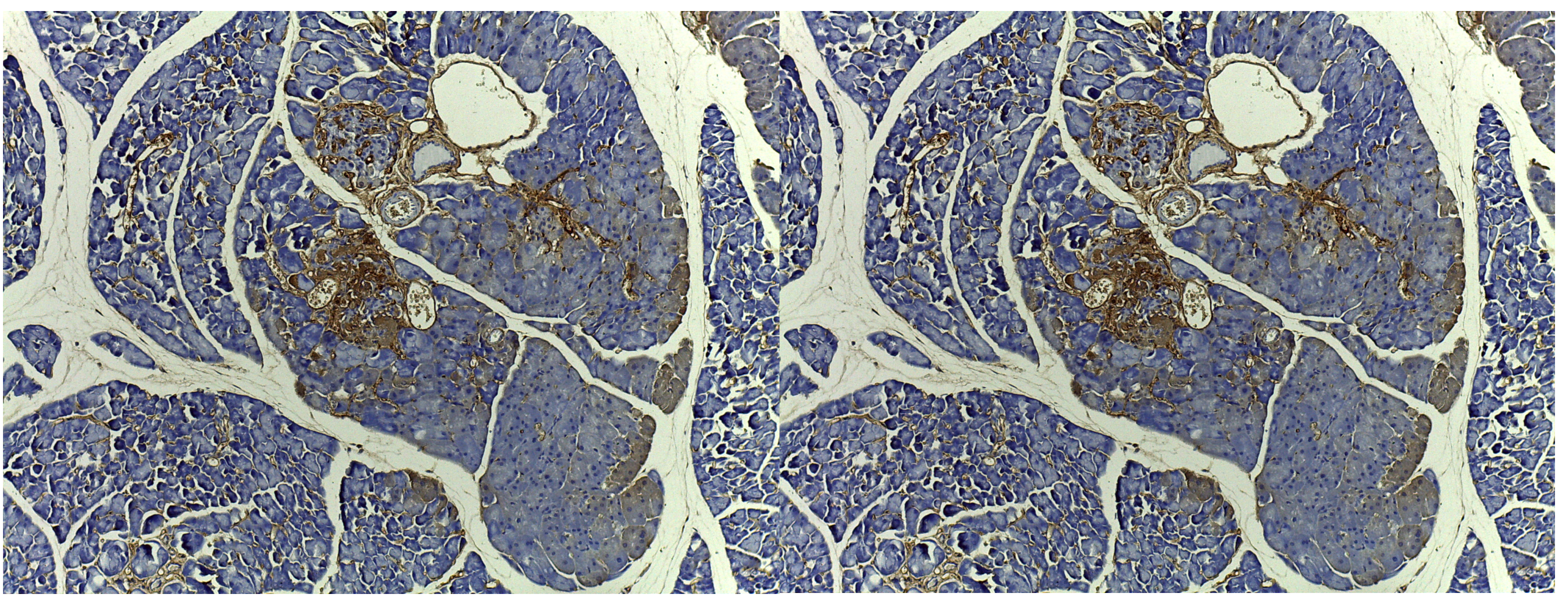

Caspase-3-N-100 Caspase-3-T-100

Westernblot

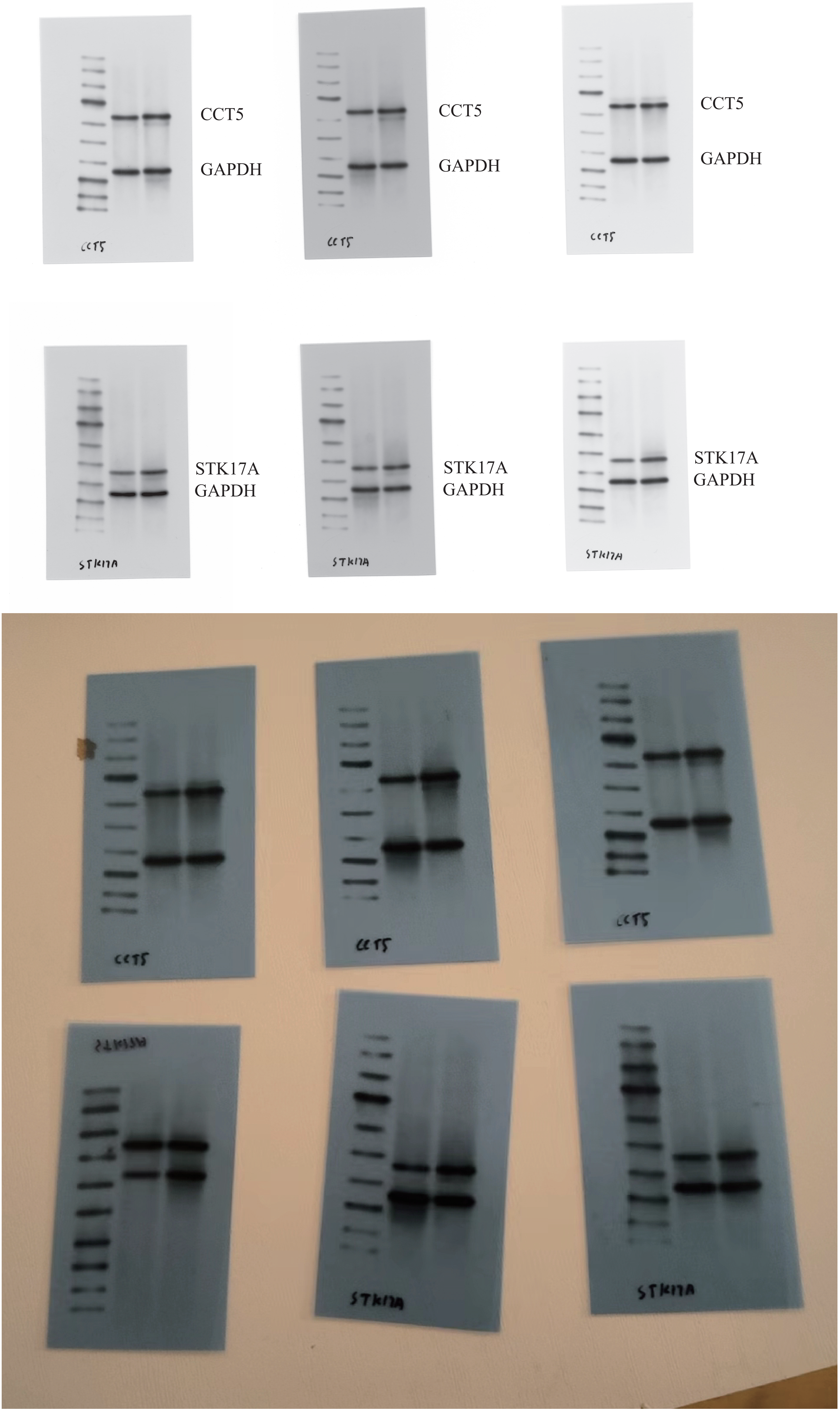

Supplement: Supplementary file 1 — Supplementary Information. [file 41598_2024_55199_MOESM1_ESM.pdf]
